# Cloud fraction response to aerosol driven by nighttime processes

**DOI:** 10.1073/pnas.2509949122

**Published:** 2025-11-21

**Authors:** Geoffrey Pugsley, Edward Gryspeerdt, Vishnu Nair

**Affiliations:** ^a^Department of Physics, Imperial College London, London, SW7 2BX, United Kingdom

**Keywords:** clouds, aerosols, climate

## Abstract

Aerosol–cloud interactions remain one of the largest uncertainties in the anthropogenic forcing of the climate; a significant contribution to this is due to the aerosol effect on the development of cloud fraction and liquid water path in stratocumulus clouds. Stratocumulus are strongly modulated by the diurnal cycle, but many previous observational studies have primarily focused on the daytime behavior of these clouds. In this work, a Lagrangian framework is used to characterize the day-night variation in the cloud sensitivity to aerosol. It is shown that the cloud fraction response to aerosol is driven by nighttime processes, whereas aerosols play a lesser role in daytime cloud fraction breakup. The liquid water path response reveals that aerosols act to thin the cloud during the daytime; however, this effect is partially offset by other processes during the nighttime. These nighttime cloud processes play an important role in setting the cloud state at the start of the day and hence the daytime cloud evolution, during which stratocumulus clouds have the greatest radiative impact. Our findings are consistent with an aerosol induced suppression of precipitation that acts most effectively at night, when stratocumulus precipitation is strongest. These results highlight a requirement for nighttime observations of marine clouds and an improved representation of the diurnal cycle in model-observation comparisons, especially when assessing climate forcing and the viability of marine cloud brightening.

## Significance

The effect of airborne particulates-called aerosols-on climate is highly uncertain due to their complex interactions with clouds. A significant source of this uncertainty comes from the aerosol influence on large, low-lying clouds over the oceans, known as stratocumulus. These clouds are known to behave differently between day and night, yet most previous observational studies have focused on the daytime. This study shows that the aerosol impact on stratocumulus is strongly time-dependent, with cloud fraction changes primarily driven by nighttime processes. These results highlight the need for more observations of nighttime cloud behavior and a better representation of the diurnal cycle in models, particularly when considering the impact of marine cloud brightening.

Aerosols and their interactions with clouds represent one of the largest sources of uncertainty in the anthropogenic forcing of the climate system (1–3). A significant contribution to this uncertainty comes from aerosol–cloud interactions in marine stratocumulus clouds. This is due to their complex interactions with aerosols and the important role they play in the Earth’s energy balance (4). Stratocumulus have a strong radiative impact due to their widespread spatial coverage, being the most common cloud type on Earth by area (5), as well as their high reflectivity in the visible spectrum (6). A small increase in stratocumulus horizontal extent (∼4%) could offset the warming from a doubling of CO_2_ (7). However, the impact of aerosols on stratocumulus cloud amount remains poorly constrained in observations (2), and in global models (8).

Aerosols act as cloud condensation nuclei, and on short timescales, an increase in aerosol concentration leads to an increase in cloud droplet number concentration, $N_{d}$ (9). For a fixed liquid water path (LWP), an increase in $N_{d}$ reduces the cloud effective radius, $r_{e}$ (9). Cloud horizontal and vertical extent are also affected by changes in $N_{d}$, collectively referred to as the cloud adjustments (10). There are two main microphysical pathways through which aerosols modify the cloud amount. First, an increase in $N_{d}$ (and a corresponding decrease in $r_{e}$) suppresses precipitation, as smaller droplets have a lower coalescence efficiency (10). This results in a longer-lived cloud (11) with a greater vertical extent (12, 13). Second, an increase in $N_{d}$ enhances entrainment efficiency at the cloud-top (14). Smaller $r_{e}$ reduces droplet sedimentation, resulting in a higher concentration of cloud droplets at the cloud-top entrainment zone, and a greater depletion of liquid water from the cloud top. As a result, entrainment-induced evaporative cooling acts to reduce cloud thickness (12, 13).

There are many studies consistent with an aerosol impact on cloud fraction (CF) (15–17). However, studies disagree on the sign and magnitude of CF adjustments. This is in part due to the strong dependence of the adjustments on cloud regime (18), as well as meteorological covariations obscuring causal relationships (19). The deepening of the boundary layer, as stratocumulus clouds are advected westward in the trade winds over warmer sea surface temperatures, plays a major role in driving cloud breakup (20). However, aerosols have also been shown to alter the cloud mesoscale structure by promoting the transition of stratocumulus cloud regimes from open to closed cell (21). This is consistent with observations of an aerosol-induced delay in the breakup of the cloud deck by suppression of precipitation resulting in a longer-lived cloud with a greater CF (11, 22), although aerosols may reduce CF in some circumstances (23, 24).

The LWP adjustment to aerosols has a strong spatial and meteorological dependence (25). This has contributed to satellite studies disagreeing on the effect of aerosol on LWP (26). Observations have reported both positive and negative LWP responses to aerosols (18, 24, 27, 28). Whereas climate models have typically predicted an increase in LWP with aerosols, partly due to incompletely representing entrainment enhancement processes (27). However, more recent global climate models have shown an improved representation of entrainment (29). Recent work has also highlighted biases in satellite retrievals of LWP and $N_{d}$ that need to be considered when assessing aerosol–LWP relationships (30).

Clouds are time-dependent and the historical state of the cloud has been shown to be an important control on the processes driving its evolution (31, 32). Stratocumulus clouds, in particular, exhibit a strong diurnal cycle (23, 33), primarily due to changes in cloud top radiative cooling (CTRC) between day and night. CTRC drives the convective instability sustaining the cloud deck (34). As parcels at the top of the cloud layer cool, they sink, displacing the air below and promoting turbulent mixing with the moist surface layer (6). Therefore, greater rates of CTRC results in more efficient coupling of the cloud layer with the surface moisture below (6). CTRC varies with the diurnal cycle due to solar insolation offsetting some of the cooling at cloud top during the daytime (35). As a result, the stratocumulus cloud field typically breaks up during the daytime and then reforms during the night (36).

The daytime breakup of the cloud deck leads to the assumption that an aerosol impact slowing stratocumulus breakup should be visible here, supported by studies suggesting that aerosols can slow the transition from closed to open-celled stratocumulus (11, 21, 37–39). Despite this, previous high-resolution modeling studies have often been carried out during nocturnal conditions (40–42) or at a fixed solar zenith angle (43) in order to allow the cloud field to reach a steady state. More recent modeling studies, which have considered the impact of the diurnal cycle on aerosol-cloud interactions, find a CF response to aerosol appearing during the day (44, 45). However, other modeling studies have found little impact of the diurnal cycle on the response to aerosol perturbations (39).

Understanding cloud nighttime processes is critical to characterize the effective radiative forcing from aerosol–cloud interactions. While the shortwave impact of these clouds at night is zero, nighttime processes set the cloud state in the morning—an important control on the evolution of the cloud and its radiative effect the following day (6, 46). A diurnal dependence on the cloud response to aerosol might be expected due to nighttime increases in CTRC modifying updraft speeds (47), thereby influencing droplet activation (48), as well as enhancing stratocumulus precipitation and entrainment rates (49, 50). However, a slowing of the daytime cloud breakup through precipitation suppression is inconsistent with ground-based observations, which show low precipitation rates during daytime (so low potential for precipitation suppression), with more significant precipitation observed at night (49).

The diversity in model responses stems from a lack of strong observational constraints about the nighttime response. While the diurnal cycle of precipitation suggests that the aerosol impact on CF will also vary over the diurnal cycle, much of the observational evidence for an aerosol impact of CF has focused on daytime conditions, relying on snapshot satellite imagery from sun-synchronous satellites with fixed local overpass times (15, 16, 19, 51). Although recent observational work has shown that both the CF and LWP sensitivities to aerosol vary over the diurnal cycle (52, 53), they have focused on instantaneous aerosol–cloud relationships, which can have difficulty in isolating the causal impact of aerosols (30) and have been shown to have poor predictive ability (45). The daytime focus in observational studies is also driven by the reliance of cloud microphysics retrievals on visible channels (54), leaving the aerosol impact on the nighttime evolution of clouds largely inaccessible to previous studies.

In this study, we characterize nighttime aerosol–cloud interactions by investigating cloud development along Lagrangian trajectories (11, 31, 55–58). We avoid the requirement for nighttime cloud observations (when satellite retrievals of cloud properties are generally less certain) by characterizing cloud development based on the early morning and late afternoon properties, separating the daytime (morning to evening) from the nighttime (evening to morning) development. A Lagrangian perspective combined with geostationary satellite data provides a way of mapping cloud development over much shorter timescales than is possible using a single polar orbiting satellite (with revisit times close to a day) and is essential as air parcels travel ∼1,000 km over the stratocumulus–cumulus transition (59) and thus cannot be fully described from a purely Eulerian perspective. We use $N_{d}$ retrievals from the MODIS Terra instrument taken in the morning, due to its close match with aircraft measurements (60), to assess the aerosol impact on cloud development along trajectories. By characterizing the aerosol impact using the initial $N_{d}$ value along the trajectory, we are not affected by the confounding impact of $N_{d}$ evolution and so more clearly isolate the causal impact of aerosols on cloud (e.g., refs. 32 and 61). We then use this method to assess how the aerosol impact on CF and LWP differs across the diurnal cycle, improving our understanding of cloud processes, identifying observational requirements and providing guidance for the timing of future marine cloud brightening deployments.

## Results

### Cloud Fraction Development.

**Fig. 1.**
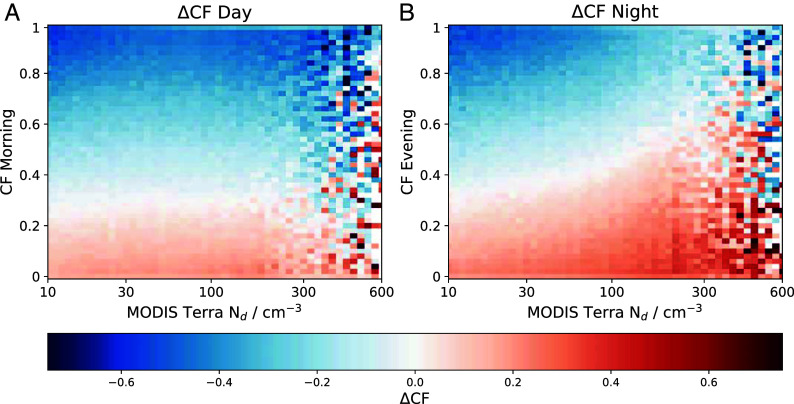
Changes in cloud fraction (ΔCF) over the daytime (evening–morning; *A*), and nighttime (morning–evening; *B*), binned by the initial CF (morning and evening respectively) and initial $N_{d}$ measured by the first MODIS Terra overpass (∼10:30 LST) in both cases, due to the lack of afternoon/early evening $N_{d}$ retrievals. Because of an issue with the heat pipe on the satellite, the morning and evening CF states are defined to be 09:00 and 18:00 LST, respectively.

Changes in CF (ΔCF) over the daytime (Fig. 1*A*) and nighttime (Fig. 1*B*) are binned based on the initial $N_{d}$ and CF (Fig. 1). Over both the day and the nighttime, clouds that start with a high CF typically decrease and those with a low CF increase. This is consistent with a “regression to the mean” effect (62). During the daytime, clouds with an initial CF of less than 0.3 tend to show an increase in CF (ΔCF > 0) across all initial $N_{d}$ conditions. A similar pattern is observed at low $N_{d}$ during the nighttime. However, at higher $N_{d}$ (≳30 cm^−3^), ΔCF remains positive even at higher initial CF values, consistent with an aerosol suppression of precipitation. Since $N_{d}$ is strongly correlated with the initial CF (11, 16, 17) it is essential to isolate any aerosol impact on ΔCF from the component of the signal that results from the regression to the mean associated with the initial CF.

There are two main pathways in which the cloud might regress to its mean state. The first of which is statistical, whereby even if the clouds were static in time, a positively biased first measurement would likely be followed by a smaller second measurement. Given the strong correlation between $N_{d}$ and CF (and LWP), an initial high $N_{d}$ retrieval would be correlated with a high initial CF and hence a larger CF decrease. This would give the appearance of an $N_{d}$-induced reduction in CF, even in the absence of any $N_{d}$ impact on CF development. The second possibility is that this correlation between initial CF (or LWP) and CF development is driven by a physical effect, independent of the aerosol environment. Again, given the strong correlation between $N_{d}$ and cloud properties, this could lead to an apparent $N_{d}$ impact on cloud development.

In order to remove this signal a new variable is introduced, the adjusted change in cloud fraction (ΔCF_*adj*_). It is defined as the ΔCF for a given $N_{d}$–CF bin, minus the mean ΔCF for the corresponding initial CF (the mean along the rows of Fig. 1). This is designed to highlight gradients in ΔCF arising from variations in $N_{d}$, independent of the initial CF. Correlations between ΔCF_*adj*_ and $N_{d}$ provide information about the effect of $N_{d}$ on CF development independent of the initial CF. It is important to note that it is the gradient of ΔCF_*adj*_ with respect to $N_{d}$ that is the physically meaningful quantity, and the sign and magnitude do not have a clear physical interpretation. We show that if there was no aerosol effect on changes in the cloud state then the corresponding “adjusted changes” would be zero (*SI Appendix*, Fig. S2).

Throughout this work, $N_{d}$ is calculated from the MODIS Terra products taken in the morning, with an overpass time of ∼10:30 LST, as it compares well to aircraft measurements (60). Parcels with a high $N_{d}$ at a given time are likely to maintain elevated $N_{d}$ values along the trajectory, as inferred from the strong correlation between $N_{d}$ measurements on subsequent days (*SI Appendix*, Fig. S1). Additionally, this work aims to investigate the impact of an air parcel’s history on its development, to better understand the causal effect of changes in $N_{d}$ on the cloud’s future state.

**Fig. 2.**
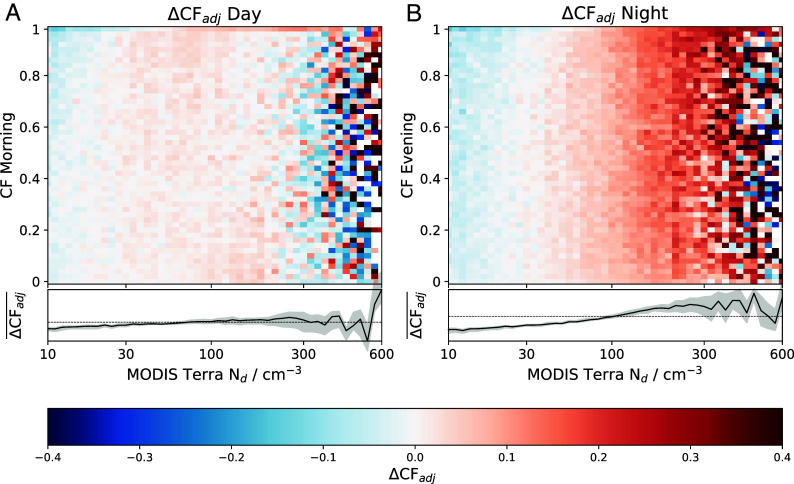
As Fig. 1, but showing the adjusted cloud fraction (ΔCF_*adj*_) change over the daytime (*A*) and nighttime (*B*) as a function of initial CF and ${\text{N}}_{d}$. The solid black line running underneath shows the mean (denoted with an over-bar) ΔCF_*adj*_ for a given MODIS Terra ${\text{N}}_{d}$. Uncertainties (shaded region) represent the 95% CI estimated using a bootstrap method with 10,000 samples (63). The mean position of the solid black line is shown as a horizontal dotted black line on the *Bottom* panel as a reference value.

Across both the daytime and nighttime, ΔCF_*adj*_ increases with larger $N_{d}$ (Fig. 2). However, a much weaker signal is seen in ΔCF_*adj*_ during the daytime (Fig. 2*A*) compared with the nighttime (Fig. 2*B*), highlighted by the black line running underneath each of the plots. This suggests that changes in CF over the daytime can be explained using other meteorological factors, while aerosol information (in the form of $N_{d}$) is needed to adequately characterize nighttime stratocumulus ΔCF.

**Fig. 3.**
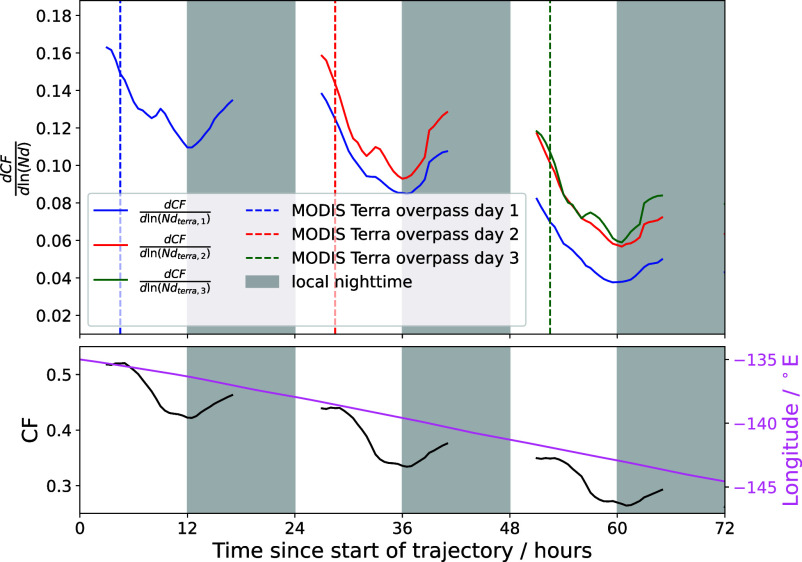
The $N_{d}$-CF sensitivity plotted over the three days of the trajectory. $N_{d}$ observations are taken from the MODIS Terra overpass at the start of the day (dashed lines). The colors (blue, red, green) indicate the $N_{d}$-CF sensitivity that would have been inferred using $N_{d}$ retrievals from the MODIS Terra overpass on day one, two and three respectively, while the CF is determined using GOES observations at each local solar time. Local nighttime is defined as ∼6pm to 6am LST. Times for which the GOES ABI instrument suffers from a heat pipe issue have been omitted. The mean CF and longitude is shown in the panel below to provide some climatological context.

The $N_{d}$–CF sensitivity (defined as $\frac{\mathrm{dCF}}{dln(Nd)}$) is calculated using a linear regression (2, 53) for different times since the Lagrangian trajectories were initialized (Fig. 3). The sensitivity exhibits strong diurnal variation, with a marked decrease after the time of the MODIS Terra overpass (∼10:30 LST). This highlights the limitations of a single snapshot overpass in capturing the full diurnal evolution of the $N_{d}$–CF relationship. It also demonstrates that it is nighttime processes that are acting to increase the strength of the $N_{d}$–CF relationship.

A general weakening of the $N_{d}$–CF relationship across subsequent days for a given MODIS overpass is observed (Fig. 3). This could be due to the fact that as the parcels are advected further westward in the trade winds over warmer sea surface temperatures, the cloud layer becomes more decoupled from the surface moisture supply and hence the CF is more strongly controlled by the reduction in atmospheric stability. This mechanism would be driven by the spatial position of the parcels and is consistent with previous results that aerosol-induced increases in cloud lifetime are greatest under stable atmospheric conditions (11) Another possible explanation is uncertainties in the Lagrangian advection: as time increases, biases in the windfield accumulate, leading to inaccurate trajectory locations and reducing the reliability of aerosol–cloud relationships over extended periods. This mechanism would be primarily a function of time. However, it is shown that small errors in the Lagrangian tracking are unlikely to significantly impact the results (*SI Appendix*, Fig. S6).

From Fig. 3, it is observed that the MODIS overpass choice has an effect on the observed strength of the sensitivity. In particular, between 24 and 36 h since the trajectory was initialized, the $N_{d}$–CF relationship inferred from the first (blue) and second (red) overpasses have broadly the same shape but with an almost constant offset. This suggests that the CF on day two is more sensitive to the MODIS Terra $N_{d}$ observation on day two than day one. However, this effect appears to be less pronounced on the third day (between 48 and 72 h), where the $N_{d}$–CF sensitivity inferred from the second and third overpasses are nearly identical. This may be due to a change in the strength of the $N_{d}$ impact on CF as the trajectory evolves, but further observation and modeling studies are required to determine the drivers of this effect.

### LWP Development.

**Fig. 4.**
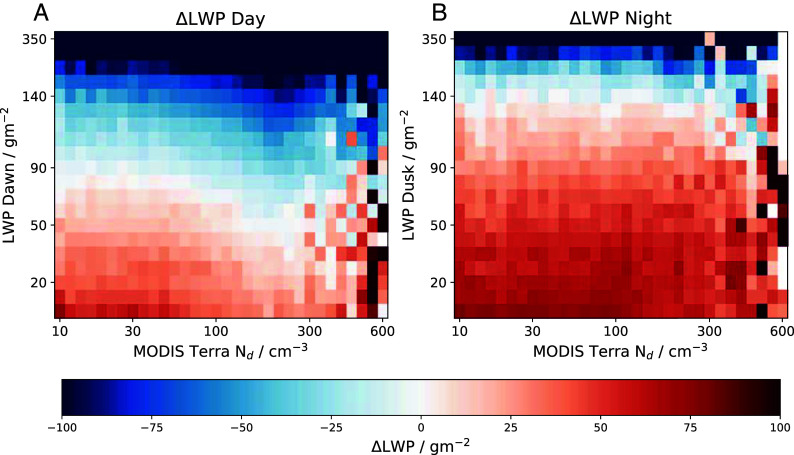
Changes in liquid water path (ΔLWP) over the daytime (*A*) and nighttime (*B*). In bins of the initial LWP and initial $N_{d}$ measured by the first MODIS Terra overpass. The morning and evening LWP states are defined to be 06:00 and 18:00 LST, respectively, such that they are symmetrical about noon.

Similar to ΔCF, the change in LWP (ΔLWP) displays “reversion to the mean” type behavior, with the largest LWP values typically decreasing regardless of $N_{d}$ (Fig. 4). At night, the LWP increases across most of the initial LWP bins under almost all conditions. This is consistent with greater turbulent mixing in the boundary layer during the nighttime promoting increases in LWP across a broad range of meteorological regimes (49). During the daytime however, a more regime dependent ΔLWP is observed (Fig. 4). For low $N_{d}$≲ 30 cm^−3^, ΔLWP changes from negative to positive close to 90 gm^−2^, however at larger $N_{d}$ this transition occurs at lower initial LWP.

**Fig. 5.**
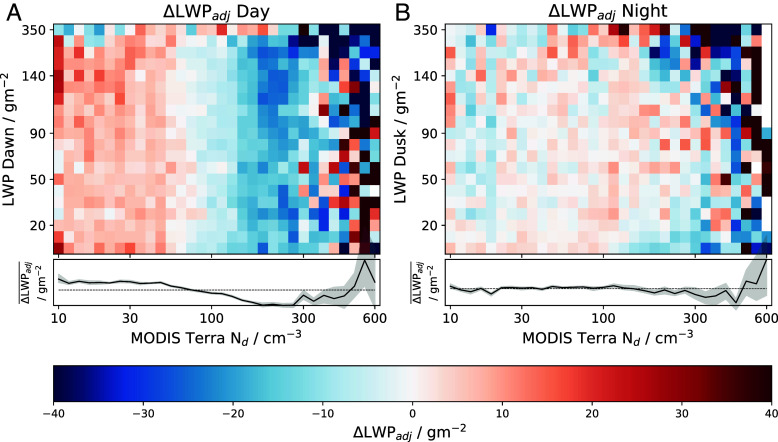
Changes in adjusted liquid water path (ΔLWP_*adj*_) over the daytime (*A*) and nighttime (*B*). In bins of the initial LWP and initial N_d_ measured by the first MODIS Terra overpass. The black line underneath shows the average ΔLWP_*adj*_ for a given $N_{d}$ bin. Uncertainties (shaded region) represent the 95% CI estimated using a bootstrap method with 10,000 samples (63). The mean position of the solid black line is shown as a horizontal dotted black line on the *Bottom* panel as a reference value.

As with CF, ΔLWP_*adj*_ is defined as the change in LWP for a given $N_{d}$–LWP bin, minus the mean ΔLWP for a given initial LWP. From Fig. 5, ΔLWP_*adj*_ shows a much steeper decrease with $N_{d}$ during the daytime compared with the nighttime, illustrated by the lines below the plots. Over the daytime ΔLWP_*adj*_ decreases steadily with increasing $N_{d}$ (between 10 and 300 cm^−3^), suggesting that aerosol is acting to thin the cloud, consistent with entrainment enhancement dominating the daytime response (52). However, during the nighttime the ΔLWP_*adj*_ response to $N_{d}$ is much flatter between 10 and 100 cm^−3^, suggesting that for lower $N_{d}$ there is a different process offsetting the daytime decrease. However, for $N_{d}$ in the range 100 to 300 cm^−3^, decreases in ΔLWP_*adj*_ with increasing $N_{d}$ are observed (which is similar to the daytime). The day and nighttime differences could be attributed to changes in precipitation over the diurnal cycle. It is shown that precipitation rates are greater during the nighttime (∼a factor of two) for this region (*SI Appendix*, Fig. S7). This is inline with previous studies (49, 64–66); hence it is during the nighttime that precipitation suppression is likely to be most significant. Therefore, it could be that precipitation suppression during the nighttime is offsetting some of the entrainment enhancement at lower $N_{d}$.

## Discussion and Conclusion

This work investigates differences in cloud responses to aerosol over the diurnal cycle, with a particular focus on cloud evolution over the day and night. Dividing the diurnal cycle into daytime and nighttime allows for the separation of two distinct CTRC regimes. This division also acts as a proxy for cloud formation and breakup processes, since stratocumulus clouds typically form during the nighttime and breakup during the day.

The aerosol effect on cloud lifetime and cloud fraction is unclear (11, 24, 67). It has been observed that during the day clouds in regions of higher aerosol abundance undergo a greater reduction in CF between morning and afternoon (23). This has been attributed to enhanced entrainment drying in higher aerosol environments during the daytime (23). However, since it is known that $N_{d}$ is correlated to CF (16), it is important to ensure that the greater reduction in CF for high $N_{d}$ is not a reversion to the mean effect. In order to achieve this ΔCF_*adj*_ was defined.

The CF generally increases during the nighttime due to enhanced CTRC and decreases during the daytime (49). This work (Fig. 2) indicates that aerosols play an important role in stratocumulus CF build-up in the nighttime and a lesser role in the breakup of the stratocumulus deck. This indicates that it is the formation processes in the nighttime that drive the $N_{d}$–CF relationship. A similar effect is seen in Fig. 3, where it is observed that the $N_{d}$–CF relationship builds up during the nighttime and then decreases again during the daytime.

Differences in ΔCF_*adj*_ are observed between the day and nighttime (Fig. 2), with a more pronounced increase in ΔCF_*adj*_ with $N_{d}$ at night compared with the daytime. Many previous studies have observed strong correlations between aerosol and cloud lifetime (11, 21) with precipitation suppression often used to describe this (10, 21, 22, 37, 39). Our results support the hypothesis that aerosol act to increase CF, with the important caveat that the aerosol impact is primarily realized during the night, consistent with the higher observed nighttime precipitation rates (49, *SI Appendix*, Fig. S7). The smaller daytime sensitivity to $N_{d}$ suggests a possible cancellation of aerosol effects, or that daytime changes are driven by effects other than $N_{d}$. This could be due to the boundary layer deepening during the daytime (68), resulting in a greater degree of decoupling between the cloud layer and surface moisture supply, leading to insolation driving cloud breakup during the day (49, 69). While greater rates of CTRC during the nighttime will also increase the rate of entrainment at the cloud top (49, 70), Fig. 2 suggests that the impact of $N_{d}$ on the nighttime CF evolution is dominated by precipitation suppression. Although during the nighttime the stratocumulus cloud radiative effect is small, with no short-wave contribution and small long-wave warming (around 15% of the daytime shortwave effect) (6), it is during these times that the aerosol effect on CF is largest. This influence of $N_{d}$ on CF during the nighttime sets the $N_{d}$–CF relationship during the daytime since it is nighttime processes that determine the cloud state in the morning and hence throughout the day. While retrieval biases can have an impact on the interpretation of aerosol–cloud relationships, an analysis with synthetic data shows these results cannot be replicated without a varying impact of $N_{d}$ on cloud development over the diurnal cycle (*SI Appendix*).

The LWP evolution displays some key differences to the CF (Fig. 4). During the daytime, ΔLWP_*adj*_ decreases with increasing $N_{d}$, whereas the nighttime response is significantly weaker with decreases only seen at higher $N_{d}$ (∼100 to 300 cm^−3^). This contrasts with the increase seen in ΔCF_*adj*_ across the day and nighttime. The negative daytime relationship is consistent with an entrainment enhancement effect on LWP due to $N_{d}$. The weakening of this signal during the nighttime suggests that a precipitation suppression effect is acting to offset some of the enhanced entrainment, assuming that these two processes dominate the cloud response to aerosol. It has been shown that there are situations where increased cloud water content through precipitation suppression can enhance entrainment at cloud top—due to increased cloud-top cooling resulting in greater boundary layer turbulence and promoting the mixing of dry air from above the cloud (13). These results suggest that the effect of the increased cloud lifetime dominates in this case. The enhanced cloud growth (from both CF and LWP) during the nighttime, relative to the daytime, is again consistent with a stronger precipitation suppression due to aerosols during the night. This is in contrast to the “inverted-V” response seen in previous studies (26, 29, 32), suggesting that the inverted-V may be a combination of daytime and nighttime relationships over the history of the cloud (52). This may further explain why model simulations suggest the $N_{d}$-LWP relationship is not a good predictor of future cloud development (45) compared to the current $N_{d}$ (Fig. 5).

Overall, this work finds a much stronger CF response to aerosol during the nighttime compared with the daytime. This is supported by a strengthening of the $N_{d}$–CF relationship during the night. $N_{d}$ is observed to thin the cloud most effectively (reducing the LWP) during the daytime, and throughout the nighttime reductions in LWP due to $N_{d}$ are only observed for higher $N_{d}$. This is consistent with a precipitation suppression effect that is stronger during the night than during the daytime, in agreement with in situ and ground-based remote sensing observations of precipitation rates (49). The observed strong diurnal signal in the cloud response to aerosol implies that the timing of potential marine cloud brightening initiatives will be crucial for their efficacy. Future observational work is needed to measure the relative strength of aerosol induced precipitation suppression and entrainment enhancement over the day and night to better relate cloud macrophysical adjustments to processes over the diurnal cycle. This will be crucial to better represent the behavior of clouds in models and will help to place more accurate observational constraints on the aerosol forcing.

## Materials and Methods

For this study, an initial domain off the coast of eastern California was selected. The domain is a square box with corners positioned at [140 ^°^ W, 130 ^°^ W] and [25 ^°^ N, 35 ^°^ N]. This location was chosen as it is a region of high stratocumulus cloud coverage, due to the strong atmospheric stability and large scale subsidence (6). Additionally, it is close to the central point on the geostationary satellite domain (GOES West CONUS), which minimized the number of cases for which a parcel was advected outside the geostationary satellite field of view. Many previous studies have also focused on this region allowing for easier comparison of this work with previous work (11, 21, 46, 71).

The initial domain is split up into a grid of 160 square cells, each with a side length of 0.25 ^°^, these will hereafter be referred to as the “parcels.” Using a Lagrangian method, the locations of the parcels along trajectories were calculated by advecting the parcel in the horizontal boundary layer (1,000 hPa) ERA5 reanalysis wind field, following the method used in previous studies (32, 72). The parcel is advected for a three day period which is the typical timescale over which the stratocumulus to cumulus transition takes place (20) and inline with previous studies (11, 52, 73, 74). Trajectories were initialized daily at 06:00 local solar time every day for the 4-y period (2019 to 2022) such that ∼2 million individual trajectories were analyzed.

Throughout this study the liquid water cloud fraction, hereafter referred to as CF, is calculated at 0.25 ^°^ using the cloud top phase mask provided by the advanced baseline imager (ABI) onboard the GOES-17 satellite (75). The CF was calculated as the fraction of pixels in a 0.25 ^°^ box that were liquid water cloud. Pixels containing ice cloud are excluded from the analysis. It is known that due to an issue with the cooling system on the instrument, the ABI IR data are degraded due to saturation. Saturation of the detector can occur between 08:30 and 17:30 UTC (76). However it has been shown that the GOES IR, from which the cloud top phase is derived, is well calibrated to other satellites outside of these times (77). When the change in the CF during daytime or the nighttime is calculated in this study the morning observation is taken at 09:00 local solar time (18:00 UTC) and the evening observation is taken at 18:00 local solar time (03:00 UTC) such that all observations are outside of the sensor saturation times. The corresponding change in CF during the daytime would be $\Delta {\text{CF}}_{\text{daytime}}=\;\text{CF}\text{(18:00 LST)}-\;\text{CF(09:00 LST)}$. The corresponding change in CF over the nighttime is the morning observation minus the previous evenings observation, e.g., $\Delta {\text{CF}}_{\text{nighttime}}=\;\text{CF(09:00 LST)}-\;\text{CF(18:00}\;{\text{LST}}_{\text{previous day}})$.

In this study the $N_{d}$–CF and LWP component of the cloud adjustment is considered, since focusing on $N_{d}$ rather than aerosol optical depth has been shown to reduce the effect of meteorological covariations by providing a causal pathway between aerosol and cloud properties (16). $N_{d}$ is calculated at pixel resolution using the standard MODIS effective radius (2.1 μm band) and cloud optical thickness following the equation in ref. 16. It is then aggregated to 1 ^°^ using the sampling strategy from ref. 78 as implemented in ref. 60. $N_{d}$ retrievals are collocated with the trajectories by selecting the closest 1 ^°^ pixel to the trajectory position at the time of the MODIS Terra overpass. 1 ^°^× 1 ^°^ cells with no valid MODIS retrievals are excluded from the analysis. The $N_{d}$ observation is taken at ∼10:30 LST, which is close to the time of maximum cloud amount (6, 79), maximizing the fraction of points with a valid mean $N_{d}$ retrieval. The impact of using the 1.6 and 3.7 μm channels for the $r_{e}$ retrievals on the results in the text is investigated in *SI Appendix* and it is shown that the different channels do not qualitatively changed when different channels are used.

The LWP data come from the gridded Remote Sensing Systems microwave LWP product on a 0.25 ^°^ grid (80–82), colocated with the trajectories. Microwave LWP retrievals are used to avoid the solar zenith angle biases identified in the GOES LWP product (83). For LWP retrievals, the morning and evening states were defined at 06:00 and 18:00 local solar time respectively, as they are not prone to the same saturation affecting the CF retrievals. Compared to optical retrieval methods microwave retrievals provide a much more direct measure of the LWP (84), making them insensitive to the solar viewing geometry (85).

Microwave retrievals of LWP cannot differentiate between liquid water droplets and precipitation and are therefore less reliable under precipitating conditions (84). Additionally, microwave imagers measure LWP including both cloud and clear sky since the spatial resolution of the sensor is much coarser than individual cloud elements (86). This results in a “beam-filling error” leading to a positive bias in the LWP retrievals, which has been corrected for in the RSS retrieval algorithm such that the LWP for clear sky scenes averages to zero (84). Despite these limitations, microwave retrievals are a useful way of measuring cloud LWP over the entire diurnal cycle.

The impact of retrieval errors on this work is described in *SI Appendix*. It is found that retrieval artifacts are negligible when the measurement uncertainty on CF is low (≲3%), which is the measurement regime that we are in (*SI Appendix*).

## Supplementary Material

Appendix 01 (PDF)

## Data Availability

The MODIS *N*_*d*_ data can be downloaded from https://catalogue.ceda.ac.uk/uuid/864a46cc65054008857ee5bb772a2a2b/ (87). The GOES-17 CONUS cloud top phase data, from which the CF is calculated, can be downloaded from https://console.cloud.google.com/storage/browser/gcp-public-data-goes-17 (88). The microwave data LWP is available at https://www.remss.com (89). The 1,000 hPa wind data came from the Copernicus Climate Change Service (C3S) Climate Data Store (CDS) (https://cds.climate.copernicus.eu/datasets/reanalysis-era5-single-levels) (90). The data and plotting code required to reproduce the figures in the paper is stored at: https://doi.org/10.5281/zenodo.17425643 (91).

## References

[1] O. Boucher , *Clouds and Aerosols* (Cambridge University Press, Cambridge, UK, 2013), pp. 571–657.

[2] N. Bellouin , Bounding global aerosol radiative forcing of climate change. Rev. Geophys. **58**, e2019RG000660 (2020). 10.1029/2019RG000660 PMC7384191 32734279

[3] P. Forster , “The earth’s energy budget, climate feedbacks, and climate sensitivity” in *Contribution of Working Group I to the Sixth Assessment Report of the Intergovernmental Panel on Climate Change in Climate Change 2021: The Physical Science Basis*, V. Masson-Delmotte , Eds. (Cambridge University Press, Cambridge, United Kingdom/New York, NY, 2021), pp. 923–1054.

[4] A. Sorooshian , A multi-year data set on aerosol-cloud-precipitation-meteorology interactions for marine stratocumulus clouds. Sci. Data **5**, 180026 (2018). 29485627 10.1038/sdata.2018.26 PMC5827690

[5] C. Hahn, S. Warren, A gridded climatology of clouds over land (1971–1996) and ocean (1954–2008) from surface observations worldwide (NDP-026E)* (2007). 10.3334/CDIAC/cli.ndp026e. Accessed 1 December 2024.

[6] R. Wood, Stratocumulus clouds. Mon. Weather Rev. **140**, 2373–2423 (2012).

[7] D. A. Randall, J. A. Coakley, C. W. Fairall, R. A. Kropfli, D. H. Lenschow, Outlook for research on subtropical marine stratiform clouds. Bull. Am. Meteor. Soc. **65**, 1290–1301 (1984).

[8] E. Gryspeerdt , Surprising similarities in model and observational aerosol radiative forcing estimates. Atmos. Chem. Phys. **20**, 613–623 (2020). 33204244 10.5194/acp-20-613-2020 PMC7668122

[9] S. Twomey, Pollution and the planetary albedo. Atmos. Environ. **8** (1251–1256), 1974 (1967).

[10] B. A. Albrecht, Aerosols, cloud microphysics, and fractional cloudiness. Science **245**, 1227–1230 (1989). 17747885 10.1126/science.245.4923.1227

[11] M. W. Christensen, W. K. Jones, P. Stier, Aerosols enhance cloud lifetime and brightness along the stratus-to-cumulus transition. Proc. Natl. Acad. Sci. U.S.A. **117**, 17591–17598 (2020). 32661149 10.1073/pnas.1921231117 PMC7395436

[12] R. Pincus, M. B. Baker, Effect of precipitation on the albedo susceptibility of clouds in the marine boundary layer. Nature **372**, 250–252 (1994).

[13] R. Wood, Cancellation of aerosol indirect effects in marine stratocumulus through cloud thinning. J. Atmos. Sci. **64**, 2657–2669 (2007).

[14] C. S. Bretherton, P. N. Blossey, J. Uchida, Cloud droplet sedimentation, entrainment efficiency, and subtropical stratocumulus albedo. Geophys. Res. Lett. **34**, L03813 (2007).

[15] B. S. Grandey, P. Stier, T. M. Wagner, Investigating relationships between aerosol optical depth and cloud fraction using satellite, aerosol reanalysis and general circulation model data. Atmos. Chem. Phys. **13**, 3177–3184 (2013).

[16] E. Gryspeerdt, J. Quaas, N. Bellouin, Constraining the aerosol influence on cloud fraction. J. Geophys. Res. Atmos. **121**, 3566–3583 (2016).

[17] D. Rosenfeld , Aerosol-driven droplet concentrations dominate coverage and water of oceanic low-level clouds. Science **363**, eaav0566 (2019). 30655446 10.1126/science.aav0566

[18] E. Gryspeerdt, P. Stier, D. G. Partridge, Satellite observations of cloud regime development: The role of aerosol processes. Atmos. Chem. Phys. **14**, 1141–1158 (2014).

[19] J. Quaas, B. Stevens, P. Stier, U. Lohmann, Interpreting the cloud cover—Aerosol optical depth relationship found in satellite data using a general circulation model. Atmos. Chem. Phys. **10**, 6129–6135 (2010).

[20] I. Sandu, B. Stevens, On the factors modulating the stratocumulus to cumulus transitions. J. Atmos. Sci. **68**, 1865–1881 (2011).

[21] D. Rosenfeld, Y. J. Kaufman, I. Koren, Switching cloud cover and dynamical regimes from open to closed Benard cells in response to the suppression of precipitation by aerosols. Atmos. Chem. Phys. **6**, 2503–2511 (2006).

[22] Y. J. Kaufman, I. Koren, L. A. Remer, D. Rosenfeld, Y. Rudich, The effect of smoke, dust, and pollution aerosol on shallow cloud development over the Atlantic Ocean. Proc. Natl. Acad. Sci. U.S.A. **102**, 11207–11212 (2005). 16076949 10.1073/pnas.0505191102 PMC1182178

[23] N. Meskhidze , Exploring the differences in cloud properties observed by the Terra and Aqua MODIS Sensors. Atmos. Chem. Phys. **9**, 3461–3475 (2009).

[24] J. D. Small, P. Y. Chuang, G. Feingold, H. Jiang, Can aerosol decrease cloud lifetime? Geophys. Res. Lett. **36**, L16806 (2009).

[25] Q. Han, W. B. Rossow, J. Zeng, R. Welch, Three different behaviors of liquid water path of water clouds in aerosol–cloud interactions. J. Atmos. Sci. **59**, 726–735 (2002).

[26] E. Gryspeerdt , Constraining the aerosol influence on cloud liquid water path. Atmos. Chem. Phys. **19**, 5331–5347 (2019).

[27] T. Michibata, K. Suzuki, Y. Sato, T. Takemura, The source of discrepancies in aerosol–cloud–precipitation interactions between GCM and A-Train retrievals. Atmos. Chem. Phys. **16**, 15413–15424 (2016).

[28] D. T. McCoy , Aerosol midlatitude cyclone indirect effects in observations and high-resolution simulations. Atmos. Chem. Phys. **18**, 5821–5846 (2018).

[29] J. Mülmenstädt , General circulation models simulate negative liquid water path-droplet number correlations, but anthropogenic aerosols still increase simulated liquid water path. Atmos. Chem. Phys. **24**, 7331–7345 (2024).

[30] A. Arola , Aerosol effects on clouds are concealed by natural cloud heterogeneity and satellite retrieval errors. Nat. Commun. **13**, 7357 (2022). 36446763 10.1038/s41467-022-34948-5 PMC9708656

[31] G. S. Mauger, J. R. Norris, Meteorological bias in satellite estimates of aerosol–cloud relationships. Geophys. Res. Lett. **34**, L16824 (2007).

[32] E. Gryspeerdt, F. Glassmeier, G. Feingold, F. Hoffmann, R. J. Murray-Watson, Observing short-timescale cloud development to constrain aerosol–cloud interactions. Atmos. Chem. Phys. **22**, 11727–11738 (2022).

[33] P. Hignett, Observations of diurnal variation in a cloud-capped marine boundary layer. J. Atmos. Sci. **48**, 1474–1482 (1991).

[34] D. K. Lilly, Models of cloud-topped mixed layers under a strong inversion. Q. J. R. Meteorol. Soc. **94**, 292–309 (1968).

[35] Y. Zheng, D. Rosenfeld, Y. Zhu, Z. Li, Satellite-based estimation of cloud top radiative cooling rate for marine stratocumulus. Geophys. Res. Lett. **46**, 4485–4494 (2019).

[36] M. Blaskovic, R. Davies, J. B. Snider, Diurnal variation of marine stratocumulus over San Nicolas Island during July 1987. Mon. Weather Rev. **119**, 1469–1478 (1991).

[37] I. Koren, G. Feingold, Aerosol-cloud-precipitation system as a predator-prey problem. Proc. Natl. Acad. Sci. U.S.A. **108**, 12227–12232 (2011). 21742979 10.1073/pnas.1101777108 PMC3145706

[38] T. Yamaguchi, G. Feingold, J. Kazil, Stratocumulus to cumulus transition by drizzle. J. Adv. Model. Earth Syst. **9**, 2333–2349 (2017).

[39] T. Goren, J. Kazil, F. Hoffmann, T. Yamaguchi, G. Feingold, Anthropogenic air pollution delays marine stratocumulus breakup to open cells. Geophys. Res. Lett. **46**, 14135–14144 (2019).

[40] F. Hoffmann, F. Glassmeier, T. Yamaguchi, G. Feingold, Liquid water path steady states in stratocumulus: Insights from process-level emulation and mixed-layer theory. J. Atmos. Sci. **77**, 2203–2215 (2020).

[41] F. Glassmeier , Aerosol-cloud-climate cooling overestimated by ship-track data. Science **371**, 485–489 (2021). 33510021 10.1126/science.abd3980

[42] G. Feingold, T. Goren, T. Yamaguchi, Quantifying albedo susceptibility biases in shallow clouds. Atmos. Chem. Phys. **22**, 3303–3319 (2022).

[43] A. Seifert, T. Heus, R. Pincus, B. Stevens, Large-eddy simulation of the transient and near-equilibrium behavior of precipitating shallow convection. J. Adv. Model. Earth Syst. **7**, 1918–1937 (2015).

[44] S. Michael , Cloud adjustments from large-scale smoke-circulation interactions strongly modulate the southeastern Atlantic stratocumulus-to-cumulus transition. Atmos. Chem. Phys. **22**, 12113–12151 (2022).

[45] J. Zhang, Y.-S. Chen, T. Yamaguchi, G. Feingold, Cloud water adjustments to aerosol perturbations are buffered by solar heating in non-precipitating marine stratocumuli. Atmos. Chem. Phys. **24**, 10425–10440 (2024).

[46] R. Eastman, R. Wood, Factors controlling low-cloud evolution over the eastern subtropical oceans: A Lagrangian perspective using the a-train satellites. J. Atmos. Sci. **73**, 331–351 (2016).

[47] Y. Zheng, D. Rosenfeld, Z. Li, Quantifying cloud base updraft speeds of marine stratocumulus from cloud top radiative cooling. Geophys. Res. Lett. **43**, 11407–11413 (2016).

[48] G. McFiggans , The effect of physical and chemical aerosol properties on warm cloud droplet activation. Atmos. Chem. Phys. **6**, 2593–2649 (2006).

[49] C. D. Burleyson, S. P. de Szoeke, S. E. Yuter, M. Wilbanks, W. Alan Brewer, Ship-based observations of the diurnal cycle of Southeast Pacific marine stratocumulus clouds and precipitation. J. Atmos. Sci. **70**, 3876–3894 (2013).

[50] D. Painemal, X. Kuan-Man, R. Palikonda, P. Minnis, Entrainment rate diurnal cycle in marine stratiform clouds estimated from geostationary satellite retrievals and a meteorological forecast model. Geophys. Res. Lett. **44**, 7482–7489 (2017).

[51] J. Quaas, O. Boucher, N. Bellouin, S. Kinne, Satellite-based estimate of the direct and indirect aerosol climate forcing. J. Geophys. Res. Atmos. **113**, D05204 (2008).

[52] K. M. Smalley, M. D. Lebsock, R. Eastman, Diurnal patterns in the observed cloud liquid water path response to droplet number perturbations. Geophys. Res. Lett. **51**, e2023GL107323 (2024).

[53] S. Qiu, X. Zheng, D. Painemal, C. R. Terai, X. Zhou, Daytime variation in the aerosol indirect effect for warm marine boundary layer clouds in the eastern North Atlantic. Atmos. Chem. Phys. **24**, 2913–2935 (2024).

[54] T. Nakajima, M. D. King, Determination of the optical thickness and effective particle radius of clouds from reflected solar radiation measurements. Part I: Theory. J. Atmos. Sci. **47**, 1878–1893 (1990).

[55] S. A. Klein, D. L. Hartmann, J. R. Norris, On the relationships among low-cloud structure, sea surface temperature, and atmospheric circulation in the summertime Northeast Pacific. J. Clim. **8**, 1140–1155 (1995).

[56] G. S. Mauger, J. R. Norris, Assessing the impact of meteorological history on subtropical cloud fraction. J. Clim. **23**, 2926–2940 (2010).

[57] R. Eastman, R. Wood, C. S. Bretherton, Time scales of clouds and cloud-controlling variables in subtropical stratocumulus from a Lagrangian perspective. J. Atmos. Sci. **73**, 3079–3091 (2016).

[58] M. W. Christensen , Evaluation of aerosol-cloud interactions in E3SM using a Lagrangian framework. Atmos. Chem. Phys. **23**, 2789–2812 (2023).

[59] I. Sandu, B. Stevens, R. Pincus, On the transitions in marine boundary layer cloudiness. Atmos. Chem. Phys. **10**, 2377–2391 (2010).

[60] E. Gryspeerdt , The impact of sampling strategy on the cloud droplet number concentration estimated from satellite data. Atmos. Meas. Tech. **15**, 3875–3892 (2022).

[61] E. Fons, J. Runge, D. Neubauer, U. Lohmann, Stratocumulus adjustments to aerosol perturbations disentangled with a causal approach. npj Clim. Atmos. Sci. **6**, 1–10 (2023).

[62] A. G. Barnett, J. C. van der Pols, A. J. Dobson, Regression to the mean: What it is and how to deal with it. Int. J. Epidemiol. **34**, 215–220 (2005). 15333621 10.1093/ije/dyh299

[63] B. Efron, Bootstrap Methods: Another Look at the Jackknife. Ann. Stat. **7**, 1–26 (1979).

[64] C. S. Bretherton, M. E. Peters, L. E. Back, Relationships between water vapor path and precipitation over the tropical oceans. J. Clim. **17**, 1517–1528 (2004).

[65] B. Stevens , Pockets of open cells and drizzle in marine stratocumulus. Bull. Am. Meteor. Soc. **86**, 51–58 (2005).

[66] E. Serpetzoglou, B. A. Albrecht, P. Kollias, C. W. Fairall, Boundary layer, cloud, and drizzle variability in the Southeast Pacific stratocumulus regime. J. Clim. **21**, 6191–6214 (2008).

[67] A. S. Ackerman , Reduction of tropical cloudiness by soot. Science **288**, 1042–1047 (2000). 10807573 10.1126/science.288.5468.1042

[68] S. Liu, X.-Z. Liang, Observed diurnal cycle climatology of planetary boundary layer height. J. Clim. **23**, 5790–5809 (2010).

[69] C. D. Burleyson, S. E. Yuter, Patterns of diurnal marine stratocumulus cloud fraction variability. J. Appl. Meteor. Climatol. **54**, 847–866 (2015).

[70] Y. Zheng, D. Rosenfeld, Z. Li, The relationships between cloud top radiative cooling rates, surface latent heat fluxes, and cloud-base heights in marine stratocumulus. J. Geophys. Res. Atmos. **123**, 11 ,678–11,690 (2018).

[71] M. Sarkar , Observations pertaining to precipitation within the Northeast Pacific stratocumulus-to-cumulus transition. Mon. Weather Rev. **148**, 1251–1273 (2020).

[72] A. Tippett, E. Gryspeerdt, P. Manshausen, P. Stier, T. W. P. Smith, Weak liquid water path response in ship tracks. Atmos. Chem. Phys. **24**, 13269–13283 (2024).

[73] R. Eastman, I. L. McCoy, R. Wood, Wind, rain, and the closed to open cell transition in subtropical marine stratocumulus. J. Geophys. Res. Atmos. **127**, e2022JD036795 (2022).

[74] P. Prabhakaran, F. Hoffmann, G. Feingold, Effects of intermittent aerosol forcing on the stratocumulus-to-cumulus transition. Atmos. Chem. Phys. **24**, 1919–1937break (2024).

[75] NASA, GOES-R Series Data Book, Revision A [Online] (2019), 240p. https://www.goes-r.gov/downloads/resources/documents/GOES-RSeriesDataBook.pdf. Accessed 09 April 2025.

[76] NOAA GOES-R Program, GOES-R Series Product User’s Guide (PUG), Volume 4: GRB [Online] (2019). https://www.goes-r.gov/users/docs/PUG-GRB-vol4.pdf. Accessed 26 February 2025.

[77] B. Tan, J. Dellomo, R. Wolfe, A. Reth, “GOES-16 and GOES-17 ABI INR assessment” in *Earth Observing System XXIV* (SPIE, 2019), vol. 11127, pp. 290–301.

[78] D. P. Grosvenor , Remote sensing of droplet number concentration in warm clouds: A review of the current state of knowledge and perspectives. Rev. Geophys. **56**, 409–453 (2018). 30148283 10.1029/2017RG000593 PMC6099364

[79] M. A. Rozendaal, C. B. Leovy, S. A. Klein, An observational study of diurnal variations of marine stratiform cloud. J. Clim. **8**, 1795–1809 (1995).

[80] F. J. Wentz, A well-calibrated ocean algorithm for special sensor microwave/imager. J. Geophys. Res. **102**, 8703–8718 (1997).

[81] F. J. Wentz, R. W. Spencer, SSM/I rain retrievals within a unified all-weather ocean algorithm. J. Atmos. Sci. **55**, 1613–1627 (1998).

[82] F. J. Wentz, T. F. Meissner, “Algorithm theoretical basis document (ATBD) version 2, AMSR ocean algorithm” (Technical Proposal 121599A–1, Remote Sensing Systems (RSS), 2000).

[83] K. M. Kevin, M. D. Lebsock, Corrections for geostationary cloud liquid water path using microwave imagery. J. Atmos. Ocean. Technol. **40**, 1049–1061 (2023).

[84] C. W. O’Dell, F. J. Wentz, R. Bennartz, Cloud liquid water path from satellite-based passive microwave observations: A new climatology over the global oceans. J. Clim. **21**, 1721–1739 (2008).

[85] A. Walther, W. Straka, “Algorithm theoretical basis document for daytime cloud optical and microphysical properties (DCOMP)” (Tech. Rep. 64, NOAA, 2020).

[86] T. J. Greenwald, T. S. L’Ecuyer, S. A. Christopher, Evaluating specific error characteristics of microwave-derived cloud liquid water products. Geophys. Res. Lett. **34**, L22807 (2007).

[87] E. Gryspeerdt , Cloud droplet number concentration, calculated from the MODIS (Moderate resolution imaging spectroradiometer) cloud optical properties retrieval and gridded using different sampling strategies (2022). 10.5285/864a46cc65054008857ee5bb772a2a2b. Accessed 1 December 2024.

[88] GOES-R Algorithm Working Group, and GOES-R Series Program, NOAA GOES-R Series Advanced Baseline Imager (ABI) Level 2 Cloud and Moisture Imagery Products (CMIP) (2017). https://www.ncei.noaa.gov/access/metadata/landing-page/bin/iso?id=gov.noaa.ncdc:C01502. Accessed 1 December 2024.

[89] F. J. Wentz, K. A. Hilburn, D. K. Smith, Remote Sensing Systems DMSP SSMIS Daily Environmental Suite on 0.25 deg grid, Version 7, CLOUD (2012). www.remss.com/missions/ssmi. Accessed 1 December 2024.

[90] H. Hersbach , ERA5 hourly data on single levels from 1940 to present (2023). 10.24381/cds.adbb2d47. Accessed 1 December 2024.

[91] G. Pugsley, E. Gryspeerdt, V. Nair, Data and code for the plots in ‘Cloud fraction response to aerosol driven by nighttime processes’. *Zenodo*. 10.5281/zenodo.17425643. Deposited 23 October 2025. PMC12663974 41269796

