# Supplementary material for: Cloud fraction response to aerosol driven by nighttime processes
Source: Proc Natl Acad Sci U S A. 2025 Nov 21;122(47):e2509949122. doi: 10.1073/pnas.2509949122 (PMC12663974; doi:10.1073/pnas.2509949122)
Supplement: Supplementary file 1 — Appendix 01 (PDF) [file pnas.2509949122.sapp.pdf]

Supporting Information for

Cloud fraction response to aerosol driven by nighttime processes

Geoffrey Pugsley, Edward Gryspeerdt and Vishnu Nair

Geoffrey Pugsley
E-mail: g.pugsley23@imperial.ac.uk

This PDF file includes:

Supporting text
Figs. S1 to S7
SI References

Supporting Information Text

MODIS N_d correlations between subsequent days. The possible combinations of the MODIS N_d observations for two days are shown in S1. Strong correlations are seen between subsequent days, however the relationship is weaker between day 1 and 3 as expected. This confirms that if a trajectory has a high N_d at a given time it is likely to be high at other times along the trajectory, and hence using the N_d at a single instance in time along the trajectory is justified.

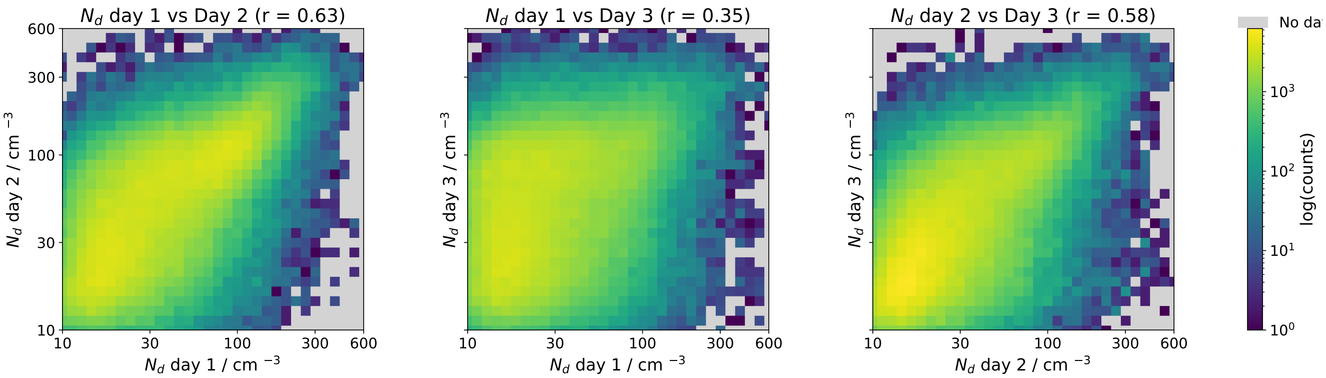

Fig. S1. N_d correlation between different MODIS overpass days

Impact of measurement uncertainties. As the aerosol-cloud system includes many complicated, interacting components, it is essential to determine whether observed relationships could have arisen without an aerosol impact on cloud evolution (1). A simple model of the system is devised here to assess this possibility.

In order to study the effect of retrieval errors on the results in the main text, theoretical true values of quantities are calculated, along with the corresponding measured value. The true values are denoted with hats, and the measured values are shown as uppercase assuming that the measurement uncertainty associated with X is distributed $\sim \mathcal{N}(0, \sigma_X^2)$. In this context X could take the values: CF, LWP or N_d such that

$$X = \hat{X} + \mathcal{N}(0, \sigma_X^2). \qquad [1]$$

A simple model is constructed assuming that a linear relationship between the log of the droplet number concentration ($log(\hat{\mathrm{N}}_d)$) and the initial cloud fraction ($\hat{\mathrm{CF}}_i$) exists according to Eq. 2.

$$\hat{\mathrm{CF}}_i = \beta \ln\left(\frac{\hat{\mathrm{N}}_d}{\hat{\mathrm{N}}_{d,0}}\right) + \hat{\mathrm{CF}}_0 + \mathcal{N}(0, \sigma_{M_1}^2) \qquad [2]$$

In equation 2, the constants $\hat{\mathrm{CF}}_0$ and $\hat{\mathrm{N}}_{d,0}$ are prescribed to be 0.75 and 300 cm^{-3} and β is 0.2; however the results are not sensitive to these choices. An initial $\hat{\mathrm{N}}_d$ field is initialised from a log-normal distribution. The corresponding $\hat{\mathrm{CF}}_i$ field is then calculated according to Eq. 2. Additional Gaussian noise ($\sigma_{M_1} = 0.2$) is added to the $\hat{\mathrm{CF}}$ field to represent variance in $\hat{\mathrm{CF}}_i$ not explained by $\hat{\mathrm{N}}_d$ (such as variations driven by other meteorological factors). The initial $\hat{\mathrm{CF}}$ field is then propagated forwards in time to calculate the CF at the end of the timestep (either dusk or dawn; $\hat{\mathrm{CF}}_f$), assuming that the $\hat{\mathrm{CF}}$ varies sinusoidally in time with a period of 1 day (2) according to Eq. 3.

$$\hat{\mathrm{CF}}_f = \hat{\mathrm{CF}}_i + a\cos(\omega t) - bt + c + \mathcal{N}(0, \sigma_{M_2}^2) + \gamma\hat{\mathrm{N}}_d \qquad [3]$$

In equation 3, (a,b,c) are set to be (0.05,0.003,-0.4) respectively. These values were chosen so that the modelled CF evolution closely resembles observations of the diurnal cycle for marine stratocumulus. The second term ($a\cos(\omega t)$) represents the diurnal variation of the CF due to changes in short wave insolation, the third term ($-bt$) produces a gradual decrease in CF over time, representing the breakup of the stratocumulus cloud deck following advection over warmer waters. Additional meteorological noise ($\sigma_{M_2} = 0.05$) is added to the CF field once it has been advanced forward in time to represent contributions to the temporal evolution not described by Eq. 3. The choice of the form the temporal development chosen for the CF does not significantly impact the results.

The final term in Eq. 3 represents the aerosol effect on $\hat{\mathrm{CF}}$ development, referred to as the *coupling term*. The magnitude of γ describes the relative strength of the N_d impact on CF evolution. A positive γ could represent a precipitation suppression effect, with $\hat{\mathrm{N}}_d$ acting to increase $\hat{\mathrm{CF}}$. Setting γ to zero removes the N_d (and hence aerosol) impact on cloud development. For this analysis γ is set to be 0.003 cm^3 whilst the coupling is turned on, and zero when coupling is false i.e. no aerosol impact on CF development.

The model representation is summarised in Fig. S2. In this analysis all changes in CF are calculated for the nighttime rather than the daytime, since it is only the effect of the coupling that is being investigated. The daytime changes would be the same as the night, but with an extra phase of π in Eq. 3.

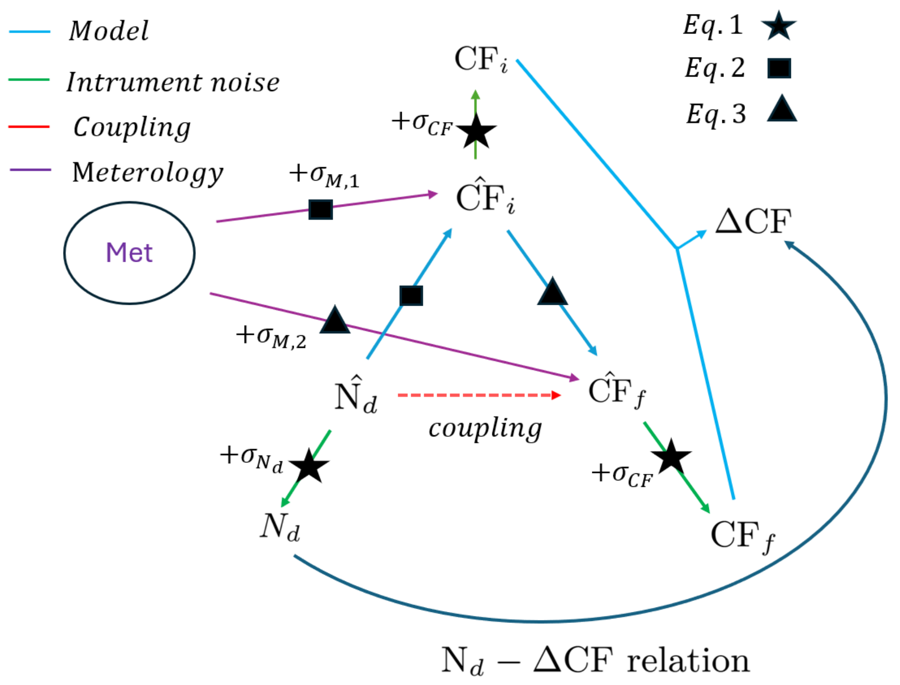

Fig. S2. The model used to study the effect of retrieval biases. Hatted symbols indicate the true value of the quantities assuming that our model (blue) and some random metrological variance (purple) fully describes these. Blue arrows indicate idealised theoretical relationships between $\hat{\mathrm{N}}_d$ and $\hat{\mathrm{CF}}$; green arrows indicate where measurement noise has been added and the dashed red arrow indicates the variable effect of N_d on CF development, this connection is not present when $\gamma = 0$. Variables in the outer triangle represent the measured satellite product. The shapes in the upper right legend reference the equation from the text that each arrow represents.

Model results. $\Delta\mathrm{CF}_{adj}$ is calculated (Fig. S3), for the cases of $\gamma = 0$ and $\gamma = 0.003\,\mathrm{cm}^3$, representing a case with no aerosol impact on CF evolution and one where aerosol acts to increase CF over time respectively. It is shown that $\Delta\mathrm{CF}_{adj}$ depends on the level of instrument noise (σ_{CF} and σ_{N_d}) as well as the strength of the coupling (γ). For low levels of CF measurement noise ($\lesssim 3\%$), when $\gamma = 0$, a very weak $\Delta\mathrm{CF}_{adj}$ is observed. Whilst when γ is non-zero significantly stronger gradients in $\Delta\mathrm{CF}_{adj}$ as N_d is varied are observed.

Recent work (3) suggests that $\sigma_{\mathrm{N}_d} \sim 65\,\mathrm{cm}^{-3}$ using the sampling strategy suggested by (4); which would correspond most closely to the second row in the figures. The GOES cloud top phase product is supplied at 2 km resolution at nadir, with an accuracy of 80 % for pixels satisfying the highest data quality flag (5). The CF is calculated at 0.25 °, therefore it would be expected that approximately 150 GOES measurements are used for each CF calculation. Combining this, the uncertainty on each CF measurement would be ∼3 %.

These synthetic data results suggest that for realistic levels of measurement noise in CF and N_d, the observed relationships between N_d and $\Delta\mathrm{CF}_{adj}$ are driven by an aerosol effect on $\Delta\mathrm{CF}$ rather than retrieval artefacts.

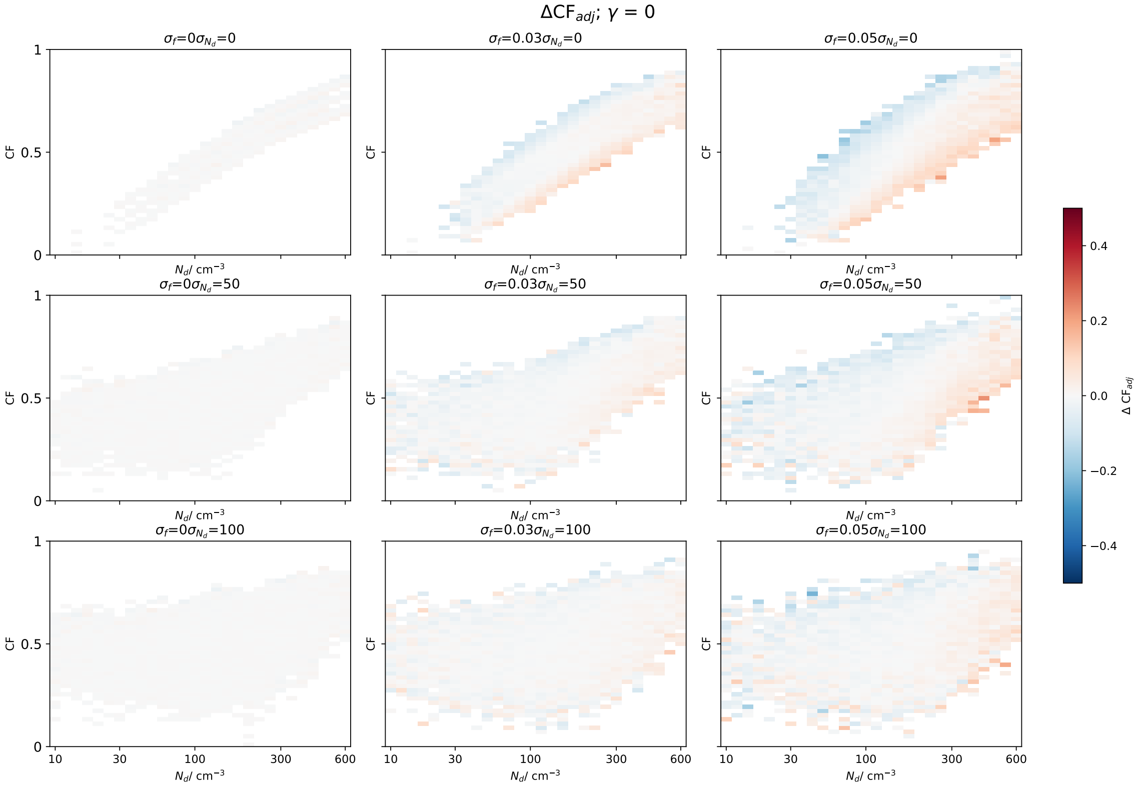

(a) Coupling off

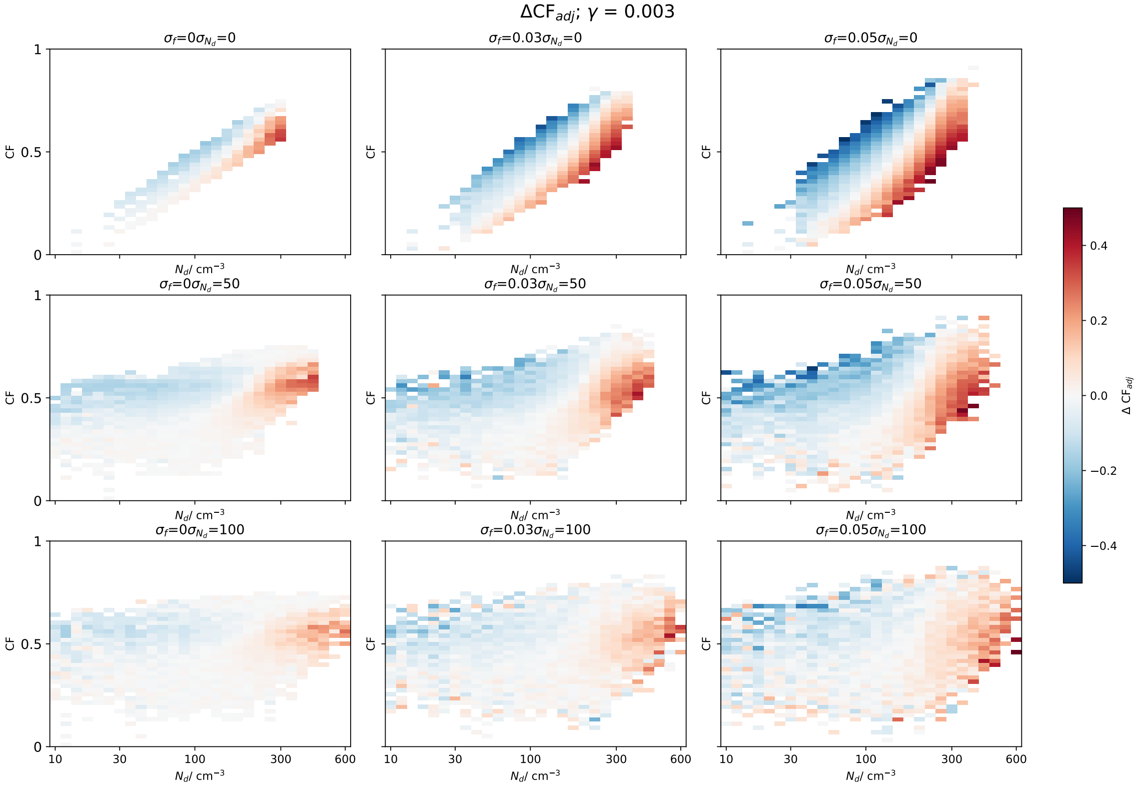

(b) Coupling on

Fig. S3. $\triangle\mathrm{CF}_{adj}$ for the nighttime with the coupling turned off (top) and on (bottom)

Sensitivity of results to MODIS channel used in r_e retrieval. The sensitivity of the main results to the choice of MODIS channel used in the r_e is investigated. The standard MODIS r_e retrieval uses the 2.6 μm channel and is used throughout the main text. It is shown that the results are qualitatively unchanged using the 1.6 μm or 3.7 μm channels (Figs. S4 and S5 respectively).

Sensitivity of the results to errors in the advection routine. It is shown that the results in the main text are not sensitive to small errors in the wind field. This is illustrated by S6 where the 850 hPa wind field was used to calculate the trajectories of the air parcels (the main text uses 1000 hPa) with little difference to the pattern of the results. Throughout this study we only consider changes over a single day or night, therefore the Lagrangian advection is only required to be accurate for up to ∼12 hours.

The diurnal cycle of precipitation. There is existing evidence of a diurnal variation in stratocumulus precipitation from the VOCALS campaign (6), which has some similarities to the region we look at in this work. Here, the diurnal variation of precipitation from the microwave retrievals in Eastman et al. (2019) (7) collocated with our trajectories is investigated. Fig. S7 provides observational evidence that both the probability of precipitation (Fig. S7 a) and rain rates (Fig. S7 b) are more likely (order a factor of two) during the nighttime. This is in line with previous studies (8–10) and supports the hypothesis that precipitation suppression is more likely to occur during the nighttime.

References

1. A Arola, et al., Aerosol effects on clouds are concealed by natural cloud heterogeneity and satellite retrieval errors. *Nat. Commun.* **13**, 7357 (2022) Publisher: Nature Publishing Group.
2. R Eastman, SG Warren, Diurnal Cycles of Cumulus, Cumulonimbus, Stratus, Stratocumulus, and Fog from Surface Observations over Land and Ocean. (2014) Section: Journal of Climate.
3. E Gryspeerdt, et al., The impact of sampling strategy on the cloud droplet number concentration estimated from satellite data. *Atmospheric Meas. Tech.* **15**, 3875–3892 (2022) Publisher: Copernicus GmbH.
4. DP Grosvenor, et al., Remote Sensing of Droplet Number Concentration in Warm Clouds: A Review of the Current State of Knowledge and Perspectives. *Rev. Geophys.* **56**, 409–453 (2018) _eprint: https://onlinelibrary.wiley.com/doi/pdf/10.1029/2017RG000593.
5. NOAA Satellite and Information Service (NESDIS), National Aeronautics and Space Administration (NASA), Goes-r series product definition and users' guide (2019) Accessed: 2025-04-10.
6. CD Burleyson, SPd Szoeke, SE Yuter, M Wilbanks, WA Brewer, Ship-Based Observations of the Diurnal Cycle of Southeast Pacific Marine Stratocumulus Clouds and Precipitation. (2013) Section: Journal of the Atmospheric Sciences.
7. R Eastman, M Lebsock, R Wood, Warm Rain Rates from AMSR-E 89-GHz Brightness Temperatures Trained Using CloudSat Rain-Rate Observations. (2019) Section: Journal of Atmospheric and Oceanic Technology.
8. CS Bretherton, ME Peters, LE Back, Relationships between Water Vapor Path and Precipitation over the Tropical Oceans. (2004).
9. E Serpetzoglou, BA Albrecht, P Kollias, CW Fairall, Boundary Layer, Cloud, and Drizzle Variability in the Southeast Pacific Stratocumulus Regime. (2008).
10. CD Burleyson, SP de Szoeke, SE Yuter, M Wilbanks, WA Brewer, Ship-Based Observations of the Diurnal Cycle of Southeast Pacific Marine Stratocumulus Clouds and Precipitation. *J. Atmos. Sci.* **70**, 3876–3894 (2013).

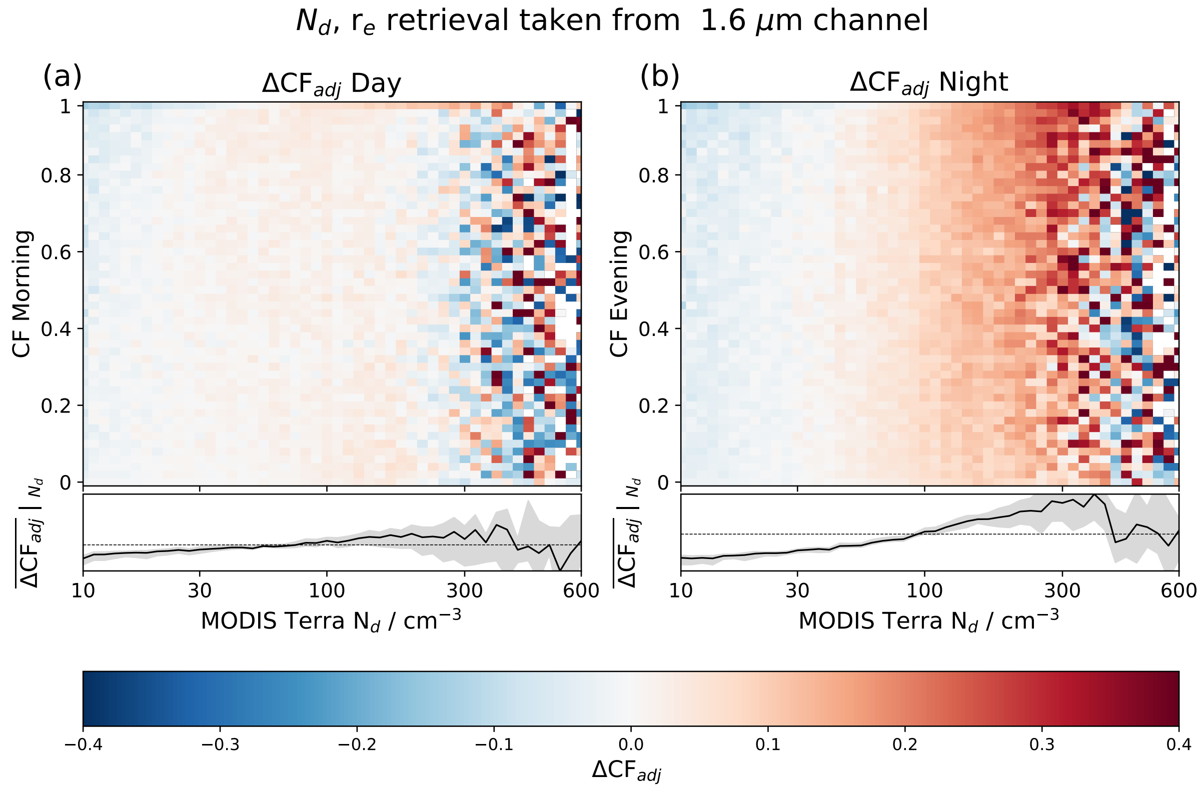

Fig. S4. As with Fig. 2 in the main text, but using the $1.6\,\mu m$ channel for the MODIS r_e retrieval used in the N_d calculation.

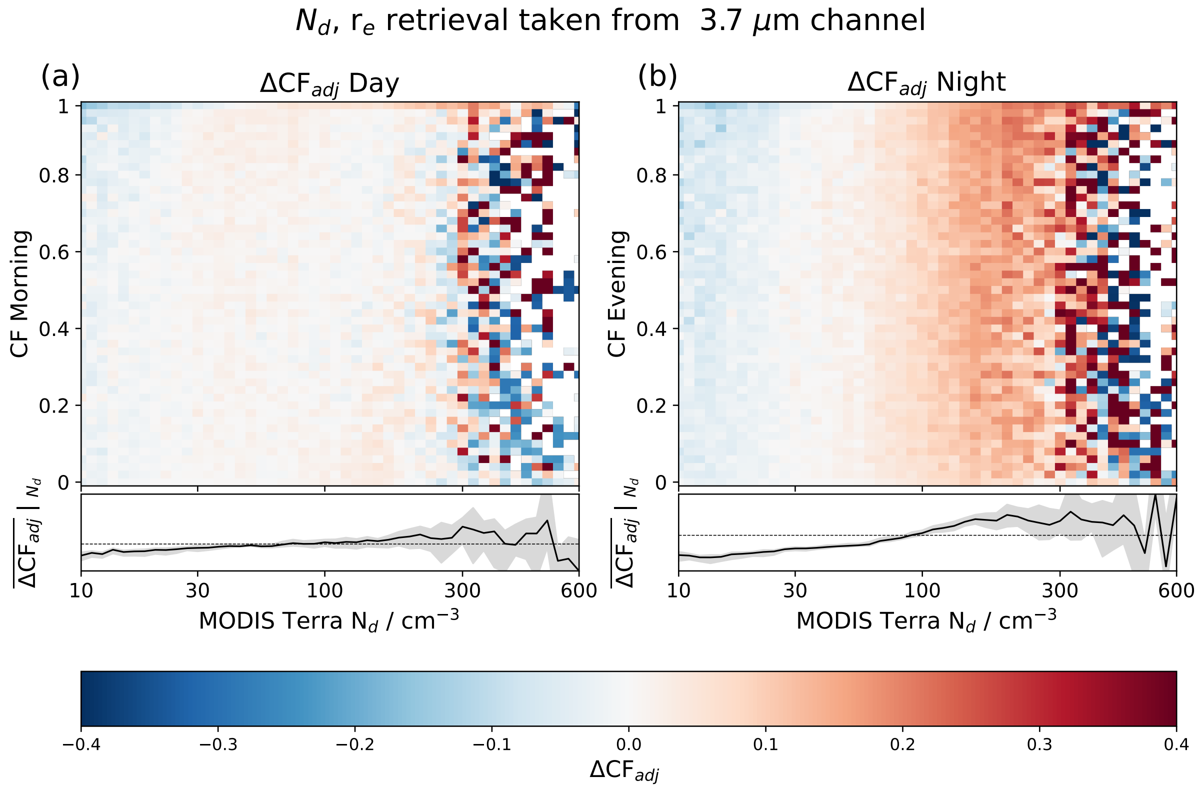

Fig. S5. As with Fig. 2 in the main text, but using the $3.7\,\mu m$ channel for the MODIS r_e retrieval used in the N_d calculation.

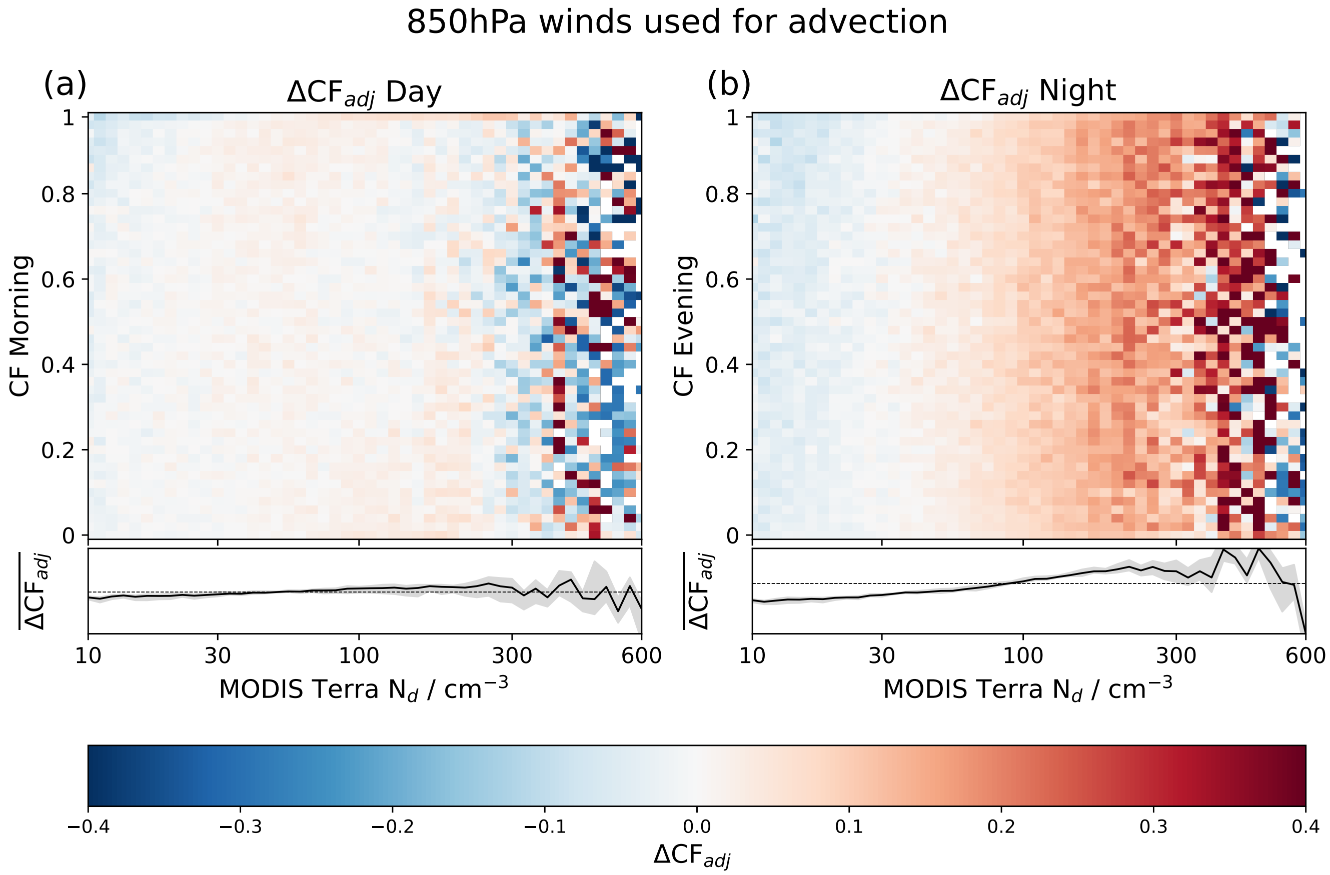

Fig. S6. As with Fig. 2 in the main text, but using the 850hPa wind field for the advection of the air parcels.

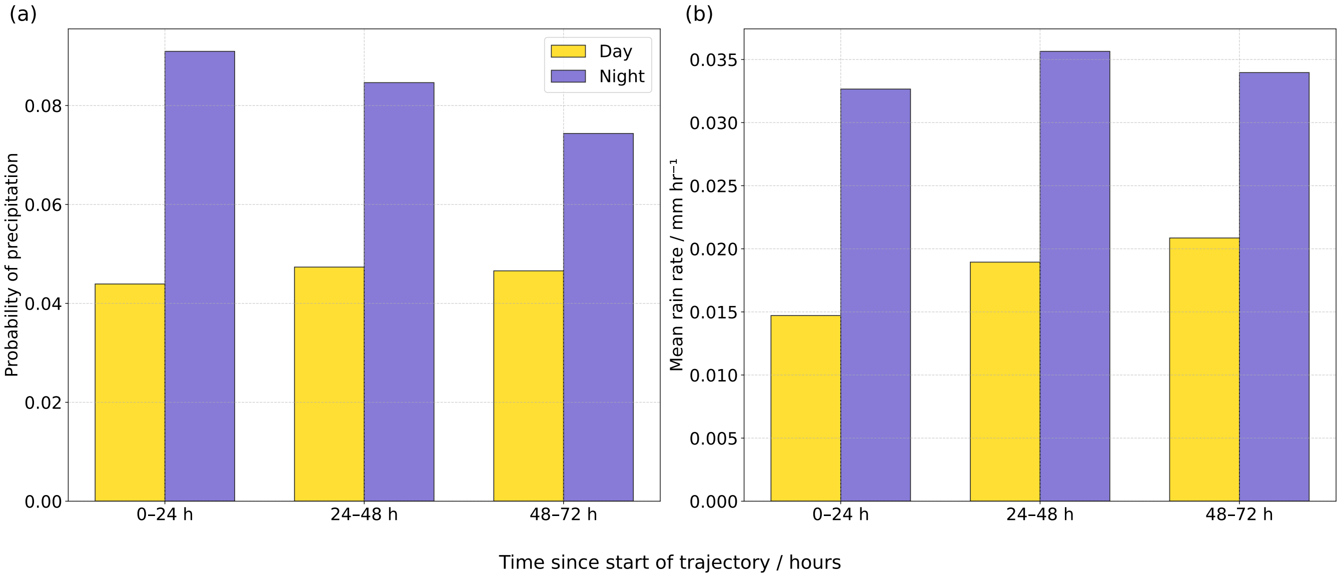

Fig. S7. AMSR warm rain rates (left) and probability of precipitation (right), co-located with the trajectories and separated by day and nighttime.
